# Biochemical Basis for the Time-of-Day Effect on Glufosinate Efficacy against *Amaranthus palmeri*

**DOI:** 10.3390/plants10102021

**Published:** 2021-09-26

**Authors:** Hudson K. Takano, Franck E. Dayan

**Affiliations:** 1 Department of Agricultural Biology, Colorado State University, Fort Collins, CO 80523, USA; 2 Mode of Action & Resistance Center of Expertise, Crop Protection Discovery and Development, Corteva Agriscience, Indianapolis, IN 46268, USA

**Keywords:** herbicide efficacy, glutamine synthetase, reactive oxygen species, enzyme turnover, mode of action, Palmer amaranth

## Abstract

Glufosinate, a glutamine synthetase (GS) inhibitor, often provides variable weed control depending on environmental conditions such as light, temperature and humidity at the time of application. Midday applications normally provide improved efficacy compared to applications at dawn or dusk. We investigated the biochemical basis for the time-of-day effect on glufosinate efficacy in *Amaranthus palmeri*. *GS1*/*GS2* gene expression and GS1/GS2 protein abundance were assessed in different parts (young leaves, old leaves, and roots) of plants incubated in the dark compared to those in the light. The turnover of GS total activity was also evaluated overtime following glufosinate treatment at midday compared to dusk application. The results suggest that GS in *A. palmeri* is less expressed and less abundant in the dark compared to in the light. Midday application of glufosinate under intense light conditions following application provide full control of *A. palmeri* plants. Consequently, these plants are unable to recover GS activity by de novo protein synthesis. Full activity of GS is required for complete inhibition by the irreversible inhibitor glufosinate. Therefore, glufosinate applications should always be performed in the middle of the day when sunlight is intense, to prevent weed escapes from the herbicide treatment.

## 1. Introduction

Glutamine synthetase (GS, E.C. 6.3.1.2) is one of the most abundant enzymes in plant cells [1]. It is essential for nitrogen metabolism because it catalyzes the conversion of glutamate and ammonium into glutamine. The two-step reaction begins with the ATP-dependent phosphorylation of glutamate into γ-glutamyl-phosphate, followed by the incorporation of ammonium and formation of glutamine [2]. Two main isoforms have been identified in different cellular compartments: GS1 functions in the cytoplasm and GS2 is compartmentalized in the chloroplast [3]. While GS1 is involved in nitrogen assimilation and transport to other parts of the plant, GS2 recycles ammonium ions generated by other physiological processes in the plant [4]. Approximately, 60% of the total ammonium consumed by GS2 come from the photorespiratory pathway [5].

The essential role of GS in plants makes the enzyme an interesting target for herbicides [6]. Inhibition of GS has catastrophic consequences to plant metabolism, leading to a massive accumulation of reactive oxygen species (ROS) due to impairment of photosynthesis and photorespiration [7]. Several experimental inhibitors have been discovered but only glufosinate has been developed as a commercial herbicide. Glufosinate is a racemic mixture of d- and l-phosphinothricin but only the l-isomer inhibits GS [8]. Studies on enzyme kinetics and crystal structure demonstrated that l-phosphinothricin is competitive with glutamate and non-competitive with respect to ammonium [9]. Inhibition of GS by glufosinate is irreversible, which is considered a suicide inhibition [2]. While GS isoforms have been extensively studied in model species, little is known about biochemical characteristics of GS isoforms in weeds. A better knowledge of such information can lead to improved weed management with GS-targeting herbicides like glufosinate.

Although glufosinate-based herbicides have been commercialized for more than 20 years, their use has increased in the last decade mainly to control glyphosate resistant weed species such as *Amaranthus palmeri* S. Watson [10]. Even with the significant increase in its use worldwide, a limited number of weeds have evolved resistance to glufosinate to date [11,12]. Moreover, genetically modified crops with resistance to glufosinate allows in-crop selective weed control as a post-emergence application [13,14]. In addition to row crops, glufosinate is also an important herbicide for perennial crops and non-agricultural areas due to its broad-spectrum activity on both monocot and dicot weed species [15].

Despite its important role as an alternative to glyphosate, glufosinate often provides a variable performance in the field. The poor performance of glufosinate in the field is often associated with its limited phloem translocation, which has been extensively studied [16,17]. Several environmental factors can influence this inconsistent response such as temperature, relative humidity, and light [18,19]. Therefore, glufosinate performance depends on the time of day at which the herbicide is sprayed in the field. Several papers have reported the time-of-day effect on glufosinate efficacy for several weed species [20,21,22]. One common observation is that glufosinate performance was lower when the herbicide was applied at either dusk or dawn compared to midday applications. While this effect has been studied for many years, little is known about the biochemical basis for such phenomenon. In this manuscript, we discuss the biochemical causes for the time-of-day effect on glufosinate performance in *A. palmeri*.

## 2. Results

**Figure 1:**
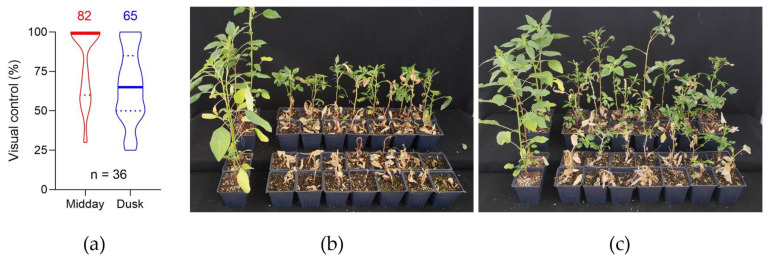
Violin plot (**a**) for % visual control of *Amaranthus palmeri* by glufosinate application (140 g ha^−1^) at midday (red) vs. dusk (blue). Pictures representing plant response to glufosinate application at midday (**b**) vs. dusk (**c**). Bold lines in the violin plot indicate the median, whereas the dashed lines represent the 95% confidence interval. Control plants are on the left side of each picture.

Visual control of *A. palmeri* plants was compared between the application of the same glufosinate dose (140 g ha^−1^) at midday (12 pm) versus at dusk (8 pm) 21 d after application (Figure 1). Glufosinate treatment provided 82% visual control on average when plants were treated at midday, and only 65% when herbicide application occurred at dusk. The median percent of control was 100% for midday application compared to 68% for dusk application. This means that most plants were controlled at 100% when glufosinate was applied at midday, indicating a more consistent performance at this time of day compared to dusk application. As indicated by the violin plot, glufosinate application at dusk, provided extremely variable control of *A. palmeri* plants, and most plants were not controlled by the herbicide sprayed at this time of day. In addition, surviving plants that were treated with glufosinate at midday were more suppressed than those treated at dusk, indicating improved weed control when applications are performed in the middle of the day.

**Figure 2:**
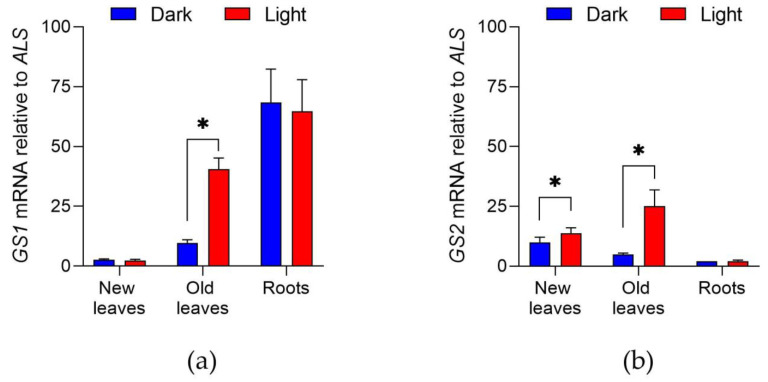
Glutamine synthetase (GS) genes are differentially expressed in the light compared to the dark depending on the plant tissue. *GS1* (**a**) and *GS2* (**b**) mRNA expression relative to ALS in new leaves, old leaves, and roots of *A. palmeri* plants growing in the light and in the dark. Asterisks indicate significant differences by t-test (*p* < 0.05) between light versus dark within each plant part.

To understand the biochemical basis for the time-of-day effect, we first investigated changes in glutamine synthetase (*GS1* and *GS2*) gene expression in different parts of an *A. palmeri* plant (Figure 2). In the shoot, we separated new leaves from old leaves because glufosinate tends to cause more phytotoxicity to older leaves than younger leaves. In general, *GS1* expression was higher in the roots compared to the shoot, whereas *GS2* expression was higher in the shoot as opposed to the roots. As expected, expression of both *GS1* and *GS2* was higher in the light, compared to dark conditions, especially for older leaves. Young leaves also had higher expression of *GS2* in the light than in the dark. Light conditions did not affect *GS1* and *GS2* expression in the root tissue.

**Figure 3:**
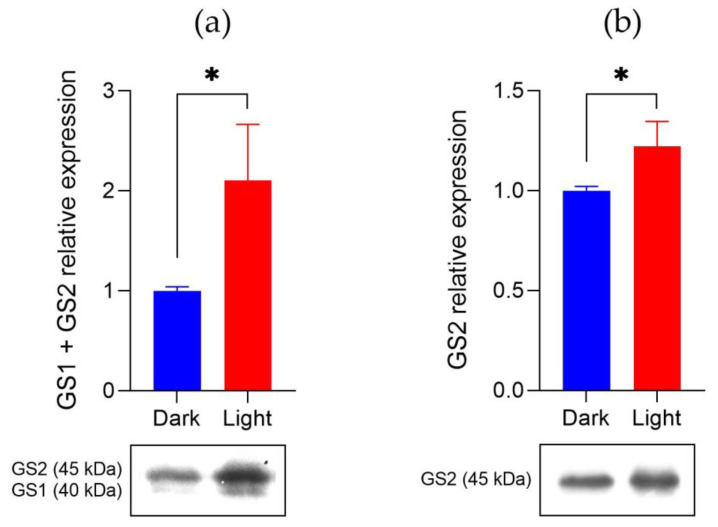
Glutamine synthetase (GS) protein abundance is higher in the light compared to dark incubated plants. Western blot protein expression analysis for both GS1 and GS2 (**a**) and for GS2 (**b**), the chloroplastic isoform of GS. Asterisks indicate significant differences by t-test (*p* < 0.05) between light versus dark conditions.

GS1 and GS2 protein expression were also compared between plants growing in the light versus those in the dark (Figure 3). Plants growing in the light showed greater abundance of both GS1 and GS2 compared to the plants in the dark. Total GS protein abundance was 2-fold higher in the light compared to the dark, whereas GS2 protein levels were only 0.3-fold greater in the light. Based on both gene and protein expression data, both GS1 and GS2 of *A. palmeri* are more expressed and abundant in the light, compared to dark conditions. Although *GS1* and *GS2* expression are lower in the dark, GS protein pools remain fairly abundant even in the dark, possibly due to residual gene expression and subsequent protein translation in the light.

**Figure 4:**
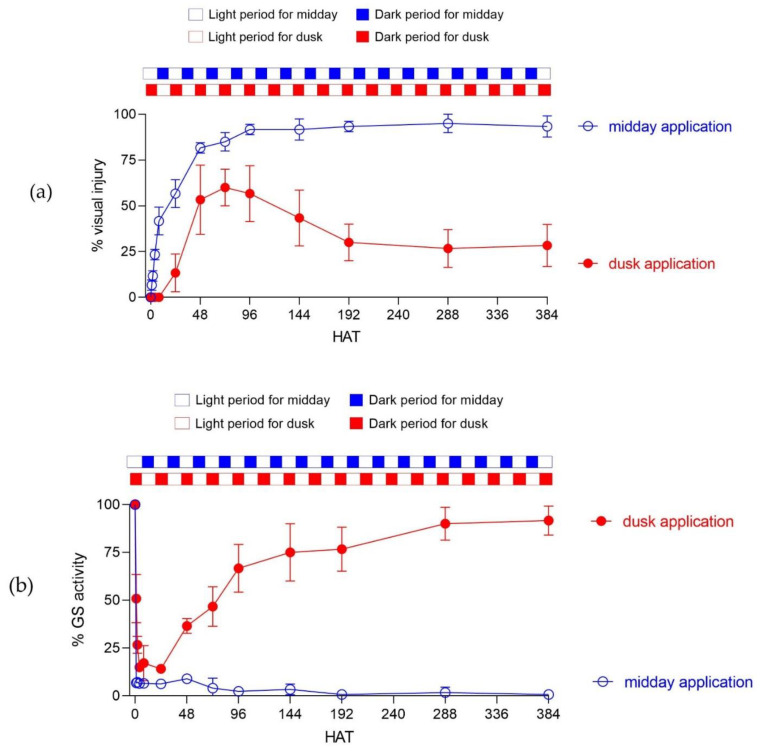
Turnover of glutamine synthetase (GS) total activity only occurs when glufosinate is applied at midday. Percent visual injury (**a**) and GS activity (**b**) after glufosinate application at midday (full red circle) or dusk (empty blue circle). Full squares represent daily photoperiod time for each application time (midday in blue, dusk in red). Dark periods are full squares whereas light periods are open squares.

Finally, we investigated the turnover of glutamine synthetase (total GS activity in the plant) in response to glufosinate treatment at midday compared to dusk application (Figure 4). For midday treated plants, the development of symptoms started two hours after herbicide application progressing exponentially overtime and reaching a constant and maximum visual injury of 95% at 96 HAT. In contrast, dusk treated plants took 12–24 h to begin showing symptoms which were much lower than those observed with the midday application. While visual injury increased up to 60% at 72 HAT, these plants treated at dusk started to recover and regrow, allowing for them to escape glufosinate application.

The turnover of glutamine synthetase following herbicide treatment provided a clear insight onto the biochemical basis for the time-of-day effect on glufosinate efficacy (Figure 4b). Glutamine synthetase activity was inhibited at >95% starting at 2 HAT in midday treated plants. This strong enzyme inhibition remained constant during the following days because glufosinate was able to maintain high levels of efficacy when application was performed at midday. In contrast, dusk application of glufosinate provided a maximum of 82% inhibition of glutamine synthetase between 2–24 HAT. After this period, plants were able to re-establish glutamine synthetase activity to the baseline levels, similar to the untreated plants.

## 3. Discussion

The time-of-day effect with glufosinate application has been known for a long time [6]. However, the biochemical basis for such observation has never been completely clear. Here, we show that the turnover of GS is responsible for glufosinate’s lower performance when applied late in the afternoon (Figure 4). We hypothesize that the plants’ ability to recover enzyme activity comes from de novo synthesis of GS, given that glufosinate was not able to completely control these plants when treatment occurred at dusk. This is particularly interesting because GS activity is known to be lower in the dark compared to the light in both *A. palmeri* (Figure 3) and other plant species [23]. In addition, *GS2* is highly expressed in the leaves while *GS1* is more abundant in roots (Figure 2). In C3 species such as rice, GS1 plays a crucial role on incorporating inorganic nitrogen into amino acids, while GS2 is a key enzyme on recycling ammonia from photorespiration [24]. Under full sunlight, plants display maximum GS activity, which keeps photorespiration ongoing. Interrupting GS activity leads to a massive light-dependent accumulation of ROS [6]. In contrast, GS activity is minimum in the dark, which makes its inhibition less damaging to photorespiration. In the dark, plants can have complete GS inhibition without apparent phytotoxicity [15].

Glufosinate is a suicide inhibitor that binds irreversibly to GS [25]. Thus, the GS enzyme pool serves as a sink for glufosinate molecules following foliar uptake even in the dark when the amount of GS protein is slightly lower than in the light (Figure 3). This “glufosinate sequestration” by GS leads to a decrease in the concentration of herbicide availability overtime. In addition, some of the glufosinate molecules may be metabolized by the plant and/or compartmentalized in the vacuole, especially when the herbicide cannot cause ROS accumulation (in the dark) [21] If the ROS-dependent rapid desiccation partially controls plants in the first days after treatment, they are able to regrow and generate more GS by de novo synthesis, allowing them to survive glufosinate treatment (Figure 4). This is what happens with dusk applications where glufosinate is unable to provide this rapid phytotoxicity following treatment because the herbicide is not active in the dark. In other words, glufosinate-treated plants at dusk can produce new GS before glufosinate can fully kill them, leading to plant survival when the herbicide is applied at dusk. Under full sunlight, however, plants suffer from a massive accumulation of ROS and rapid leaf burning. This prevents regrowth after glufosinate treatment and de novo synthesis of GS.

In conclusion, glufosinate is a light-dependent herbicide that must be applied in the hours in the middle of the day when sunlight is maximum. When glufosinate is applied in the early morning or late afternoon, or even in cloudy days, binding to GS does not lead to ROS accumulation and subsequent cellular burst with foliage desiccation. Once bound to GS, glufosinate cannot inhibit a different GS because it is a suicide inhibitor. This leads to a decreased concentration in available glufosinate molecules overtime inside the cells, which allows for plant survival and de novo synthesis of GS. Thus, glufosinate has only one chance of controlling weeds, which happens in the first few days after treatment, restricting the application to the middle hours of a sunny day for maximum performance.

## 4. Materials and Methods

### 4.1. Quantification of the Time-of-Day Effect on Glufosinate Efficacy

Seeds of Palmer amaranth (*Amaranthus palmeri* S. Watson) were collected in a corn field located in eastern Colorado, USA. Plants were grown in 0.3-cm^3^ pots filled with soil (Sun Gro Horticulture, Agawam, MA, USA) until they reached 10 cm-tall (6–8 fully expanded leaves). Environmental conditions were 25/21 C day/night, 14 h photoperiod (6 am–8 pm) with light intensity of 500 μmol m^−2^ s^−1^ and 70% relative humidity (RH). For herbicide applications, a commercial chamber track sprayer (DeVries Manufacturing, Hollandale, MN, USA) equipped with an 8002EVS single even, flat-fan nozzle (TeeJet^®^, Spraying Systems, Denver, CO, USA) calibrated to deliver 187 L ha^−1^ spray solution at the level of the plant canopy was used. Glufosinate commercial formulation (Liberty^®^ 280 g L^−1^, BASF, Raleigh, NC, USA) was applied at 140 g ha^−1^ with 2% (*w*/*v*) ammonium sulfate. Two growth chambers were used to mimic the time-of-day treatment. Applications were performed at noon (12 pm) for plants in one growth chamber, or at dusk (8 pm) in plants from the other chamber. Each treatment had 36 replications and plants were visually evaluated, where 0% means no effect and 100% plant death, at 21 d after treatment (DAT). The experiment was repeated in time. The datapoints were used to construct a violin plot which are similar to box plots, except that they also show the probability density of the data at different values.

### 4.2. GS1 and GS2 mRNA Expression in the Light vs. Dark and Different Plant Parts

Palmer amaranth plants were grown up to the 10 cm-tall growth stage as described above. “Light plants” refer to those that were sampled six hours after dawn, whereas “dark plants” were harvested six hours after dusk. Plant tissue (50 mg) was collected from new leaves, old leaves, and root tips, and immediately frozen in liquid nitrogen. The “new leaves” were considered as the two youngest leaves and the apical meristem, whereas “old leaves” were the oldest two fully expanded leaves in the plant. The tissue was ground using a Qiagen Tissuelyzer (Qiagen, Valencia, CA, USA). Total RNA was extracted using Qiagen RNeasy PlantMini Kit, treated with DNase I, quantified using a NanoDrop spectrophotometer (Thermo Scientific), and checked for quality and integrity with agarose gel electrophoresis (clear bands with no contamination or degradation, gels are not shown). Two hundred nanograms of RNA were used for synthesizing cDNA using Super Script cDNA synthesis kit (Thermo Scientific) with oligo-dT and random hexamers. Primers were designed for GS1, GS2 and acetolactate synthase (ALS) as internal standard: GS1_F (5′- AACCATGGTACGGTATCGAACAGG -3′) and GS1_R (5′- AGGCAAGCCTTGTAGTGTGAATC -3′), GS2_F (5′- AAGGATCCATTCCGTGGTGG -3′) and GS2_R (5′- TCTCAGAAACAACCTTTGGGTCG -3′) [26], and ALS_F (5′- GCTGCTGAAGGCTACGCT -3′) and ALS_R (5′- GCGGGACTGAGTCAAGAAGTG -3′). Palmer amaranth cDNA (final concentration of 0.4 ng μL^−1^) was amplified using quantitative real-time polymerase chain reaction (PCR) with 25 μL total volume under the following conditions: 10 min at 95 °C, 40 cycles of 95 °C for 20 s and 59 °C for 1 min. After completing these cycles, temperature was 59 °C increasing by 0.5 °C every 5 s until reaching 95 °C to obtain the melting curve. *GS* mRNA expression was calculated as ΔCt = (Ct for ALS—Ct for GS), and the relative increase in *GS* expression was expressed as 2ΔCt [27]. Three biological replications were tested, and each sample was run three times to calculate the mean and standard deviation. The experiment was repeated in time and data was pooled. Results were represented as the fold increase in *GS* expression relative to ALS.

### 4.3. GS1 and GS2 Protein Abundance in the Light Versus Dark

Palmer amaranth plants were grown up to the 10 cm-tall growth stage as described above. Light plants were sampled six hours after dawn, whereas dark plants were harvested six hours after dusk. Three plants of each condition were assessed. Western blot method followed protocol described elsewhere [28]. Leaf tissue (1 g) was ground in liquid nitrogen and homogenized with 2 mL extraction buffer composed of 50 mM Tris (2-amino-2-(hydroxymethyl)-l,3 propanediol, pH 8), 2 mM EDTA (ethylmediamine tetraacetic acid), 10 mM mercaptoethanol, 10% (*v*/*v*) glycerol. The crude extract was filtered through two layers of miracloth and clarified by centrifugation at 20,000 *g* for 30 min. The pellets were discarded, and the supernatants were used for Western blot analysis. All steps were conducted at 4 C. The concentration of protein was estimated by the Bradford (1976) method with bovine serum albumin as a standard. Proteins were analyzed by denaturing electrophoresis in the presence of sodium dodecyl sulphate (SDS). The stacking gel was 5% (*w*/*v*) polyacrylamide (2.5% cross-linker) and the resolving gel 12.5% (*w*/*v*) polyacrylamide (2.5% cross-linker). After electrophoresis, the polypeptides were visualized by staining for 2 h in a solution containing 1% (*w*/*v*) Coomassie blue R-250, 10% (*v*/*v*) acetic acid, 40% (*v*/*v*) methanol. The gel was extensively de-stained overnight in the same solution without the dye. Molecular masses (Mr) in kilodaltons (kDa) were estimated relative to a protein ladder (company). Polypeptides previously separated by SDS-polyacrylamide gel electrophoresis (PAGE) were transferred electrophoretically to a nitrocellulose membrane (Millipore, Bedford, Mans, USA) using a Multiphor II Novablot apparatus (LKB, Uppsala, Sweden). The electroblotting was conducted by the application of 0.8 mA. cm^−2^ constant current for 90 min, and 39 mM glycine, 48 mM Tris, 0.04% (*w*/*v*) SDS, 20% (*v*/*v*) methanol was used as transfer buffer. The blot was processed for immunolabelling by incubation for 60 min in a blocking solution containing 3% (*w*/*v*) bovine serum albumin, 0.05% (*v*/*v*) Tween-20 in phosphate-buffered saline (PBS; 140 mM NaCl, 3 mM KCl, 5 mM Na_2_HPO_4_; pH 7.4). The filter was then incubated overnight with GS1/GS2 (Agrisera, AS08295) or GS2 (Agrisera, AS08296) antibodies raised against GS from *Arabidopsis thaliana*. A 1:1000 dilution in 0.5% (*w*/*v*) bovine serum albumin, 0.02% (*v*/*v*) Tween-20, PBS, was the working solution. Finally, the nitrocellulose sheet was washed another three times with PBS and the peroxidase activity viewed by incubation in 0.02% (*v*/*v*) H_2_0_2_, 4 mM 4-chloro-1-naphthol in PBS. The enzymatic reaction was stopped by two washes with distilled water. The Western blots were wrapped in aluminum foil until photographed. The gel image was then converted to gray scale and reverted. Protein bands were quantified by measuring their relative intensity using photoshop (Adobe Photoshop, v.22.4.3).

### 4.4. The Turnover of Glutamine Synthetase Activity Following Glufosinate Treatment

*A. palmeri* plants were grown and sprayed at midday or dusk as described above. In total, six replications were used for each timepoint, and the experiment was repeated in time. Visual injury and glutamine synthetase enzyme activity were evaluated at 1, 2, 4, 12, 24, 48, 72, 96, 144, 192, 288 and 384 h after treatment (HAT). *In planta* glutamine synthetase activity was quantified by the GS-dependent formation of γ-glutamyl hydroxamate by measuring the transferase activity [29]. Similar doses, growth stage, number of replications, and spray settings from glufosinate dose response section were used for this assay. Eight HAT, 2 g of leaf material was collected and homogenized in a chilled mortar with 3 mL of extraction buffer. The extraction buffer (pH 8) consisted of 50 mM Tris base, 1 mM EDTA, 2 mM DTT, 10 mM MgCl_2_, and 50% (*w*/*v*) PVP10. The extract was then filtered through miracloth layered on top of cheesecloth after washing the mortar with additional 1 mL extraction buffer. The filtered extract was centrifuged at 12,000× *g* for 10 min at 4 °C. The reaction consisted of 0.9 mL of assay buffer and 0.1 mL of crude enzyme extract, incubated for 30 min at 30 °C. The assay buffer consisted of 25 mM imidazole–HCl (pH 7.5), 4 mM MnCl_2_, 5 mM ADP, 50 mM l-glutamine, 40 mM sodium arsenate, and 25 mM hydroxylamine. To stop the reaction, 0.5 mL ferric chloride reagent was added, and the mixture was incubated for 10 min at room temperature, followed by centrifugation at 12,000 × *g* for 10 min. The ferric chloride reagent consisted of 32% (*w*/*v*) anhydrous ferric chloride dissolved in 0.5 M HCl. The concentration of γ-glutamyl hydroxamate was determined by measuring absorbance at 540 nm on a spectrophotometer (SynergyTM 2 Multi-Mode Microplate Reader; BioTek, Winooski, VT, USA).

### 4.5. Data Analysis

Statistical analysis was performed in R software (v.4.1.0, The R Foundation) All data passed normality by visual inspection of normality plots, and homogeneity of variances by Levene’s test. The means for *GS1* and *GS2* mRNA expression and for GS1 and GS2 protein abundance were compared by t-test (P <0.05). The other data were graphed and represented as the mean ± standard deviation. All graphing were performed with Prism software (GraphPad v. 8.3.1, San Diego, CA, USA).
